# Acute Acalculous Cholecystitis in a Febrile Adolescent: A Rare Hepatobiliary Manifestation of Acute Epstein-Barr Virus Infection

**DOI:** 10.7759/cureus.90359

**Published:** 2025-08-18

**Authors:** Antreas Savvidis, Maria Anatoliotaki, Angeliki Tzagkaraki, Eirini Papamitsaki, Sofia Stefanki

**Affiliations:** 1 Pediatrics, Venizelion General Hospital, Heraklion, GRC; 2 Radiology, Venizelion General Hospital, Heraklion, GRC

**Keywords:** acute acalculous cholecystitis (aac), complication, febrile, inpatient pediatrics, primary ebv infection

## Abstract

We report a case of previously healthy 15½-year-old girl who presented with fever and vomiting. Notably, she developed mild scleral icterus and had imaging findings suggestive of acute acalculous cholecystitis (ACC). Targeted serological testing confirmed acute Epstein-Barr virus (EBV) infection. The patient improved with supportive care. This case highlights the potential for EBV to cause acute hepatobiliary pathology, emphasizes the importance of including viral etiologies in the differential diagnosis of febrile adolescents with hepatobiliary findings, and aims to raise awareness about this rare complication.

## Introduction

Epstein-Barr virus (EBV), also known as human herpesvirus 4, is a gammaherpesvirus of the *Lymphocryptovirus* genus and represents the leading cause of infectious mononucleosis, accounting for over 90% of cases. Nearly 90% of adults worldwide have evidence of prior infection. Endemic infectious mononucleosis is frequently observed in adolescent group settings, such as schools and military institutions. The classic clinical presentation includes fever, pharyngitis with petechiae or exudates, lymphadenopathy, hepatosplenomegaly, and atypical lymphocytosis [1,2]. Mild and temporary increases in liver enzymes are commonly seen in ΕBV infectious mononucleosis. Around 5% of affected individuals may also experience mild jaundice, which can be due to either impaired bile flow (cholestasis) or destruction of red blood cells caused by the virus hemolysis [3]. We present a case of acute acalculous cholecystitis (ACC) secondary to EBV-induced infectious mononucleosis, with clinical and radiographic findings supporting the diagnosis. ACC is an atypical clinical presentation of primary EBV infection [4].

## Case presentation

A 15½-year-old previously healthy girl, fully vaccinated for age, was admitted with a history of persistent fever up to 39°C, poorly responsive to antipyretics, and a sore throat. Over the preceding three days, she developed frequent non-bilious vomiting. Two days prior to admission, she visited our hospital's emergency department.

At the emergency department, initial laboratory investigations (Table 1) showed leukopenia, white blood cells (WBC) at 4,400/µL, mild thrombocytopenia of 14,5000 /µL, mild elevated inflammatory markers: C-reactive protein (CRP) at 1.2 mg/dL, erythrocyte sedimentation rate (ESR) at 44 mm/h (<10 mm/h); and elevated liver enzymes: serum glutamic-oxaloacetic transaminase (SGOT) at 169 U/L, serum glutamic pyruvic transaminase (SGPT) at 175 U/L, gamma-glutamyl transferase (γ-GT) at 203 U/L, and alkaline phosphatase (ALP) at 237 U/L. Targeted serological test was performed due to the suspicion of EBV infection. EBV serology confirmed acute primary infection: viral capsid antigen (VCA) immunoglobulin M (IgM) positive, EBV IgG negative [2]. Cytomegalovirus (CMV) and toxoplasma serologies were negative. Peripheral blood smear revealed lymphocytosis with numerous reactive lymphocytes. Blood cultures were negative. The physical examination revealed palpable hepatomegaly and splenomegaly and cervical lymphadenopathy.

**Table 1:** Laboratory test results WBC: White blood count; CRP: C-reactive protein; ESR: erythrocyte sedimentation rate; SGOT: serum glutamic-oxaloacetic transaminase; SGPT: serum glutamic pyruvic transaminase; γ-GT: gamma-glutamyl transferase; ALP: alkaline phosphatase. '-' indicates not measured.

Parameter	Reference Range	ED	Repeat Test 1	Repeat Test 2	Follow-Up (2 Weeks)
WBC (cells/µL)	4,500–11,000	4,400	6,800	6,100	3,500
Lymphocytes (cells/µL)	>1,500	2,500	4,400	4,400	1,800
Platelets (µL)	150,000–450,000	145,000	150,000	281,000	197,000
CRP (mg/dL)	<0.4	1.2	0.8	<0.4	<0.4
ESR (mm/h)	<10	44	55	84	30
SGOT (AST) (U/L)	<34	169	217	60	28
SGPT (ALT) (U/L)	10–49	175	231	82	22
γ-GT (U/L)	<38	203	228	264	98
ALP (U/L)	46–116	132	265	323	130
Amylase (U/L)	30–118	–	68	52	39
Total Bilirubin (mg/dL)	0.3–1.2	–	2.8	0.9	1.6
Direct Bilirubin (mg/dL)	<0.3	–	1.5	0.6	0.6

**Figure 1:**
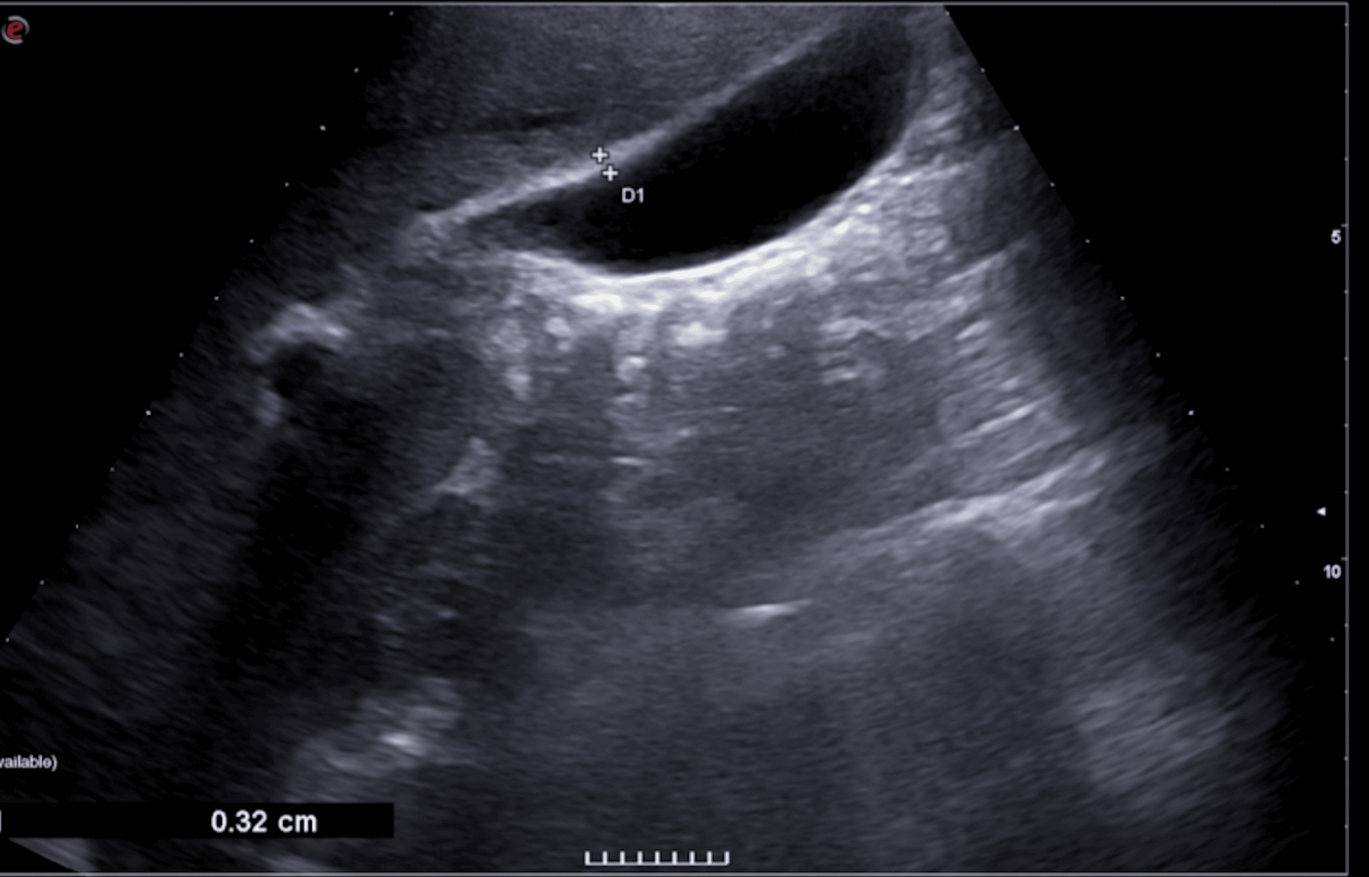
Ultrasonographic image of the gallbladder during the initial emergency department visit, which demonstrates a gallbladder with normal wall thickness (3.2 mm)

Abdominal ultrasonography showed hepatomegaly (21.24 cm craniocaudal diameter), splenomegaly (21.19 cm) [5], and a normal gallbladder with a wall thickness of 3.2 mm (Figure 1). 

The pancreas, biliary tract, and kidneys appeared normal. She was discharged from the emergency department with symptomatic treatment.

The patient returned two days later due to persistent symptoms and new-onset fatigue. On re-examination, she appeared moderately ill. Scleral icterus was noted for the first time. Bilateral cervical lymphadenopathy was observed, with the largest node measuring approximately 2 cm. The examination of the abdomen revealed a non-distended abdomen with active bowel sounds and mild epigastric tenderness. The liver was palpable 2 cm below the right costal margin, and the spleen 2 cm below the left costal margin. Right periorbital edema was also noted.

Repeat laboratory testing results presented in Table 1 showed elevated WBC at 6800/µL (4,500-11,000 cells/µL), elevated lymphocytes at 4400/µL, normal platelets at 150,000/μl, elevated inflammatory markers: CRP at 0.8 mg/dL, ESR at 55 mm/h, elevated liver enzymes: SGOT at 217 U/L, SGPT at 231, γ-GT at 228 U/L, ALP at 264 U/L, amylase at 53 U/L, total bilirubin at 2.8 mg/dL, and direct bilirubin at 1.5 mg/dL. Peripheral smear revealed lymphocytosis with 16% reactive/atypical lymphocytes. Due to the above findings, she was admitted to our department for further investigation and management.

**Figure 2:**
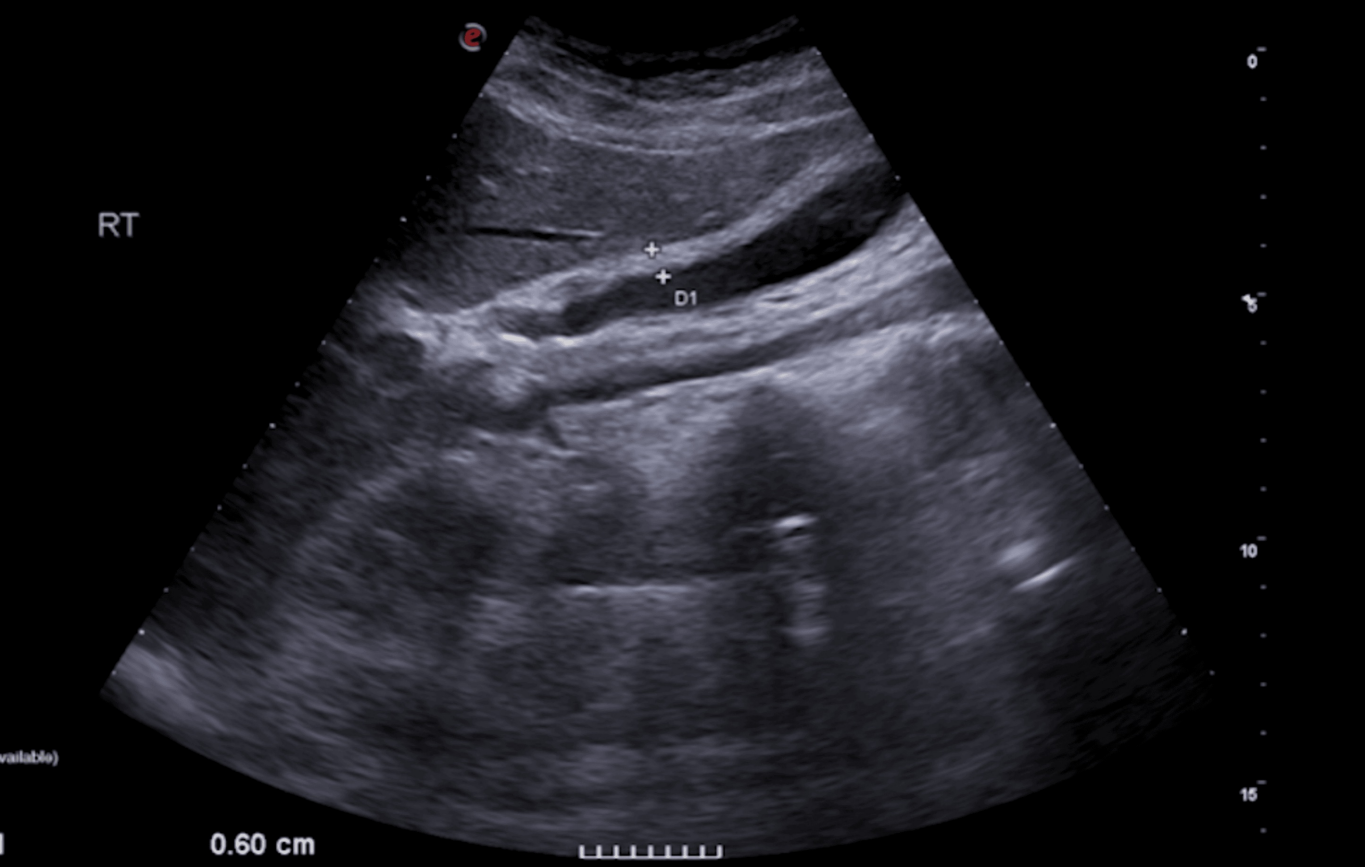
Ultrasonographic image of the gallbladder on the third day of hospitalization, showing a thickened wall (6 mm) without evidence of gallstones, suggestive of acute acalculous cholecystitis (ACC)

On the third day of hospitalization, the patient continued vomiting and exhibited right upper quadrant abdominal tenderness with a positive Murphy’s sign. The repeat ultrasound showed hepatomegaly (20.0 cm), splenomegaly (19.75 cm) [5], and gallbladder wall thickening of 6 mm without evidence of gallstones (Figure 2), raising suspicion for ACC. The combination of supportive clinical and laboratory findings, along with a gallbladder wall thickness of ≥3.5 mm, is generally considered the diagnostic of ACC [6]. A gastroenterology consultation supported this diagnosis.

Management was supportive, including intravenous hydration, and a low-fat diet was commenced. Over the next two days, the patient’s symptoms improved significantly, with resolution of vomiting, and the patient’s jaundice progressively improved.

Due to ongoing sore throat, a throat culture swab was obtained, which was positive for Group C beta-hemolytic *Streptococcus*, deemed unrelated to the cholecystitis [1,7]. It can also be a complication of the EBV infection [2]. Intravenous clindamycin was started according to antibiogram.

The patient was discharged on the 10th day of hospitalization in good clinical condition. The repeat laboratory values (Table 1) showed elevated WBC at 6100/µL, elevated lymphocytes at 4400/ µL, elevated platelets at 281,000/μL, CRP at <0.4 mg/dL, and elevated values of other parameters: ESR at 84 mm/h, SGOT at 60 U/L, SGPT at 82 U/L, γ-GT at 264 U/L, ALP at 323 U/L (46-116 U/L), amylase at 56 U/L (30-118 U/L), total bilirubin at 0.9 mg/dL, and direct bilirubin at 0.6 mg/dL.

Follow-up 

Τhe patient was examined two weeks later with new blood test (Table 1), which revealed almost normal values: WBC at 3500/µL, lymphocytes at 1800/ µL, platelets at 197,000 /μl, CRP <0.4 mg/dL, slightly elevated ESR at 30 mm/h, SGOT at 28 U/L, SGPT at 22 U/L, elevated γ-GT at 98 U/L, ALP at 130 U/L, slightly elevated total bilirubin at 1.6 mg/dL, and elevated direct bilirubin at 0.6 mg/dL. From the blood smear, few reactive lymphocytes were found. She was reevaluated three weeks after discharge. Her blood test results were normal, and a new abdominal ultrasound showed a normal gallbladder wall thickness, with the liver measuring 15.5 cm and the spleen 13.5 cm.

## Discussion

Patients with EBV infectious mononucleosis have been noted to present with isolated gallbladder wall thickening [3]. However, AAC associated with EBV infectious mononucleosis is a rare occurrence, with only a limited number of cases described in the literature [6]. AAC is uncommon in children and typically arises in association with systemic infections such as septicemia, salmonellosis, brucellosis, or hepatitis A virus infection. Diagnostic indicators include a gallbladder wall thickness exceeding 3 mm, gallbladder distention, localized tenderness, the presence of pericholecystic fluid, and sludge. A diagnosis of AAC is generally made when two or more of these features are observed in the appropriate clinical context [8]. Hepatitis caused by EBV has been identified as an important contributor to cholestasis [9]. In our patient, cholestatic hepatitis was attributable to acute EBV infection, which may indicate a potential contribution of EBV-induced cholestasis to the pathogenesis of AAC. This case underscores the importance of considering viral etiologies, including EBV, in adolescents presenting with hepatobiliary symptoms. EBV-induced hepatitis can present with cholestasis, jaundice, and even gallbladder wall thickening, causing acute cholecystitis. In our case, the absence of gallstones and improvement with conservative management helped avoid unnecessary surgical intervention.

Reactive lymphocytosis and atypical lymphocytes are hallmarks of EBV infection and supported our clinical suspicion, which was confirmed serologically. The development of scleral icterus and rising bilirubin prompted repeated imaging, which revealed findings suggestive of ACC, a rare but documented complication of EBV [6].

Our findings suggest that, beyond gallbladder wall thickening, AAC can emerge as a complication of acute EBV infection, particularly in the setting of cholestatic hepatitis. Conservative therapeutic approaches may be suitable for EBV-related acute cholecystitis; however, careful surveillance is advised.

## Conclusions

Cholecystitis in adults is most often related to gallstones, whereas infections tend to be more common causes in children. In some cases, AAC may occur in children alongside EBV infection. Many such cases respond well to conservative medical treatment, just like our patient. Diagnosing AAC early can be challenging, but clinical signs and lab results suggesting a viral infection can help raise suspicion. When ultrasound indicates features of AAC, EBV infection may be considered as part of the differential diagnosis.
